# Draft Genome Sequence of Neopoerus faecalis gen. nov., sp. nov., an *Oscillospiraceae* Strain Isolated from Human Feces

**DOI:** 10.1128/mra.00330-23

**Published:** 2023-06-21

**Authors:** Marta Selma-Royo, Liviana Ricci, Davide Golzato, Charlotte Servais, Federica Armanini, Francesco Asnicar, Federica Pinto, Nicola Segata

**Affiliations:** a CIBIO Department, University of Trento, Povo, Trento, Italy; DOE Joint Genome Institute

## Abstract

Here, we report the isolation and genome assembly of a strictly anaerobic bacterium from a previously uncharacterized species in the *Oscillospiraceae* family, isolated from a fecal sample from a healthy adult human. The name Neopoerus faecalis gen. nov., sp. nov. is proposed.

## ANNOUNCEMENT

The family *Oscillospiraceae* includes genera such as *Faecalibacterium* and *Ruminococcus* that are linked to host health (1) and are predicted to produce beneficial metabolites (2), while several representatives of this family remain to be cultivated.

We describe the isolation of an *Oscillospiraceae* strain in an anaerobic chamber (Baker Ruskinn Concept 400) (95% N_2_/5% H_2_). The fecal sample was collected from a healthy donor in November 2022 (Trento, Italy), and it was processed within the hour. Two fecal dilutions (10^−5^ and 10^−7^) were spread onto modified brain-heart infusion medium (BHIm; Fluka AG) agar plates supplemented with 2.5 g/L yeast extract (Fluka AG), 2 g/L cellobiose (Alfa Aesar), 2 g/L maltose (Sigma-Aldrich), 2 g/L soluble starch (Rozzano), 1 g/L l-cysteine (Sigma-Aldrich), 1 mg/L resazurin sodium salt (Sigma-Aldrich), 0.005% vitamin K1 (Alfa-Aesar), 5 mg/L hemin (Thermo Fisher), and 4% defibrinated sheep blood (SB, Microbiol Diagnostics). After 3 days of incubation (37°C), one single colony was inoculated in 5 ml of BHIm broth plus SB for 48h. Genomic DNA was isolated using the Wizard genomic DNA purification kit (Promega) and used for sequencing library preparation with the Illumina DNA prep and tagmentation kit. The libraries were sequenced (150-bp paired-end reads) on a NovaSeq 6000 instrument with S4 flow cell reagents (Illumina) at the University of Trento (Italy), after a cleaning step using 0.6× Agencourt AMPure XP beads.

The raw reads were preprocessed using Trim Galore (parameters: “–stringency 5 –length 75 –quality 20 –max_n 2 –trim-n”) (https://github.com/FelixKrueger/TrimGalore). A total of 7,772,460 high-quality paired-end reads (mean quality [Q] value, 35.47) were retained, with a mean read length of 148.21 bp. Genome assembly was performed using SPAdes v3.15.2 (3) (parameters: –careful -k 21,33,55,77,99,127) using 30% of the high-quality reads (https://github.com/lh3/seqtk; parameters: sample module), as they were already above 100× coverage for a putative genome of 3.5 Mb. After standard filtering using the NCBI pipeline and removal of contigs of <1,000 bp, we obtained 36 contigs spanning a total length of 2.54 Mb. The assembly and annotation statistics are reported in Table 1. The assembly statistics were computed using QUAST v5.1.0rc1 (4), and measures of completeness and contamination were obtained using the lineage_wf workflow from CheckM v1.1.2 (5). Unless specified, all the computational tools used in this work were used with default parameters.

**TABLE 1:** Summary of the statistics of the Neopoerus faecalis sp. nov. genome assembly^*a*^

Parameter	Value
Total length (bp)	2,542,847
No. of scaffolds	36
GC content (%)	56.22
Mean coverage (×)	116
Size of longest scaffold (bp)	496,649
*N*_50_ (bp)	274,696
*L*_50_	4
No. of coding sequences	2,408
No. of rRNAs	7
No. of tRNAs	57
Estimated completeness (%)	98.66
Estimated contamination (%)	0

a The genome assembly was annotated using Prokka v1.14. The assembly statistics were computed using QUAST v5.1.0rc1, and the completeness and contamination were obtained using the lineage_wf workflow from CheckM v1.1.2. The mean coverage was computed using CMSeq v1.0.4 (https://github.com/SegataLab/cmseq).

**FIG 1:**
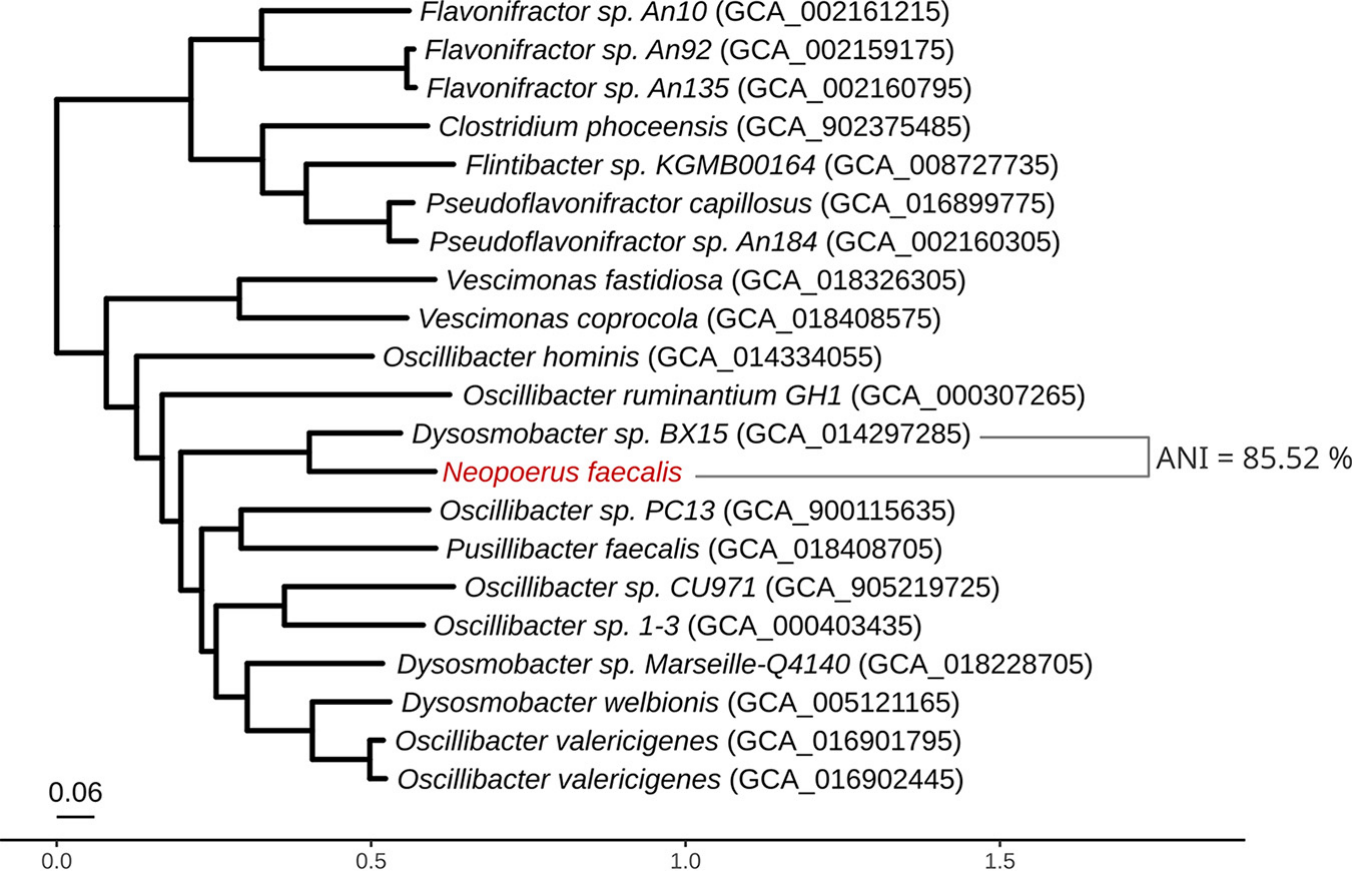
Phylogenetic tree of the *Neopoerus faecalis* gen. nov, sp. nov. isolate and related taxa with available reference genomes based on whole-genome sequencing (WGS). Sequence accession numbers are given in parentheses. The closest taxonomically defined species is Dysosmobacter welbionis (GenBank accession number GCA_005121165), with an ANI of <80%. The closest available reference genome, *Dysosmobacter* sp. strain BX15 (GCA_014297285), is mislabeled at the genus level, since it has an ANI of <85% to the *Dysosmobacter* reference strain (*Dysosmobacter welbionis*).

Annotation using Prokka v1.14 (6) revealed that the strain harbors a total of 2,475 genes. Screening for antibiotic resistance genes using the Resistance Gene Identifier v5.1.1 (7) identified resistance to tetracycline, diaminopyrimidine, and aminoglycoside. Phylogenetic analysis using the PhyloPhlAn v3.0 pipeline (January 21 version; parameters: -d phylophlan –diversity low –fast –force_nucleotides) (8) showed that the closest taxonomically defined species was Dysosmobacter welbionis (GenBank accession number GCF_005121165.3) at <80% average nucleotide identity (ANI; computed using FastANI v1.33 [9]) (Fig. 1). This genetic distance to the closest known taxa warrants the definition of a new genus and species (10). The closest reference genome available in the NCBI database was that of *Dysosmobacter* sp. strain BX15 (ANI, 85.52%; GCA_014297285), a genus-level mislabeled strain, since it shows an ANI of <85% with the *Dysosmobacter* sp. reference genome (Fig. 1), thus warranting the classification of the new strain as *Neopoerus faecalis* sp. nov. Pipeline analysis using METABOLIC (11) revealed genes related to complex carbohydrate degradation, such as cellulose and chitin. Previous surveys estimated the prevalence of Neopoerus faecalis sp. nov. at 40.6% after the mapping of its specific marker genes across more than 24,500 available metagenomes from five continents (12).

### Data availability.

This study project is available under NCBI BioProject accession number PRJNA939950 and BioSample accession number SAMN33549472. The assembly and the reads used to assemble the genome are available under GenBank accession number JARFXX000000000 and SRA accession number SRX20179702, respectively.
